# Appraisal of World Health Organization (WHO) infection prevention and control guidelines

**DOI:** 10.1186/1753-6561-5-S6-P281

**Published:** 2011-06-29

**Authors:** SP Montes, F Otaiza, M Watson, S Eremin, S Hill, CL Pessoa-Silva

**Affiliations:** 1 World Health Organization, Hanoi, Vietnam; 2 Min. Health, Santiago, Chile; 3 World Health Organization, Geneva , Switzerland

## Introduction / objectives

Several WHO guidelines related to infection prevention and control (IPC) of infections associated with health care have been issued over the past decades. A review of all available WHO IPC-related guidelines was conducted.

## Methods

All WHO IPC-related guidelines accessible through WHO website published until December 2009 were appraised, and the technical content of each document was appraised against current policies and standards promoted by WHO. *Definitions:* "active WHO document" is considered to be an official WHO document available on the WHO web site; classification of guidelines regarding alignment with current recommendations: (1) “valid”, (2) “out of date”.

## Results

87 IPC documents were evaluated, 12 non-relevant to IPC were excluded, and 31 guidelines produced by 11 Departments were identified and appraised. 13/31 (42%) guidelines were considered ‘out of date’ regarding Standard Precautions (8 documents), disinfection (6 documents), sterilization of medical equipment (6 documents), and preparedness for epidemics (3 documents). Outdated messages included ineffective and unsafe practices (e.g., disinfection of disposable sharps, fumigation of the environment with formaldehyde, use of personal protective equipment). Most ‘out of date’ guidelines (10/13) were issued before 2001 as compared to ‘valid’ ones (1/18) (P = 0.0001).

## Conclusion

The search targeted documents that are specific or closely related to the IPC field, but due to the cross-cutting nature of IPC this inventory may not include all WHO documents including IPC recommendations. The IPC field has rapidly evolved and the audience should look for most recent and evidence-based documents to guide practices.

## Disclosure of interest

None declared.

